# Illegal Medicine

**Published:** 1899-12

**Authors:** Nelson W. Wilson

**Affiliations:** Buffalo, N. Y.


### ILLEGAL MEDICINE.

Conducted by NELSON W. WILSON, M. D., Buffalo, N. Y.

TWO DEATHS FROM "CHRISTIAN SCIENCE."

DURING the past month there have been two cases of christian science which by reason of the vicious cruelty under which the patients died are of particular public interest.

Since the publication of the first matter in this department last month, I have received a letter from a physician in Buffalo, whose judgment is uniformily excellent. He commends the JOURNAL for its firm stand in all matters which are against the public good, but suggests that "too much publicity" can be given to the subject of christian science. As a general proposition in matters of dispute "too much prominence in print serves to advertise an opponent," as the correspondent says, but in the case of christian science "too much" prominence cannot be given this unholy sect. Their acts, their beliefs, their prayers, their teachings and their victims cannot be given "too much" prominence. Christian scientists are not opponents. They are evils; public evils, and as much of a menace to public health as an epidemic of the plague. They are the latter-day types of the medieval witch-doctors; they are the editions *de luxe* of the snake-skinning voodoos and charm-chanting medical men of benighted heathen nations. Repeated efforts to check their practices by legal methods have failed; not because there are not laws covering their acts, but because, unfortunately, money has been used and the influence of those in high places has been thrown into the balance at critical moments, and politics appealed to. This statement applies particularly to New York State. In Kentucky, the land of the "bloody ground," fakirs in medicine are not tolerated; but in the Empire State where a beneficent legislature says that a certain fistic artist must not punch another fistic artist in the nose outside of a regularly licensed and incorporated athletic club, and that even then he must not do his punching unless his fists are properly gloved, efforts to correct an evil which permits a pain-rocked soul to "pass on" to kingdom come without so much as a good-bye, to say nothing of an absolute lack of the slightest attempt to lessen the death agony, are met by a fierce appeal to religious rights, and politicians, angling always for class vote, side in with the scientists and either pigeonhole or destroy corrective measures.

This is made possible by the fact that christian scientists, whatever else we may say of them, are clannish. Let one christian scientist fall into the clutches of the law, and the whole body gather together ready, willing and anxious to put up money for defense. Class differences for the time being buried and all put their shoulders to the wheel and work—not for their fellow-fakir who is in the toils, but for their cause. They show the highest type of fanaticism. One of the scandals of the christian science outfit in Buffalo was said to be the quarrel between "Rev." "Dr." Hardy and one of the women followers of Mrs. Eddy's double-cross. This quarrel, so public report says, was over the instructing of young healers, and resulted in a split. When the Kinters were arrested for their connection with the Saunders case, "Rev." "Dr." Hardy and the woman in question were found side by side in the council chamber before the board of aldermen's committee on ordinances, fighting for a common cause. That is one secret of their apparent strength. The same reason accounts for the temporary strength of the followers of the Mahdi's green flag of fanaticism. The history of all fanatics is the same. The cohesive power of unity makes them strong at the outset, but their fate is the same in the end. It may be that which befell the ghost dancing Indians of our own west; it may be that which overtook the hordes of cut-throats which fell before Lord Kitchener's onslaught, and it may be the less gory, but none the less effective which will overtake the christian scientists of today, but it will be the same in effect—a crushing of a great evil.

CHRISTIAN SCIENCE AND VACCINATION.

Mayor Hixon and the city council of Americus, Georgia, has been wrestling with christian science and have set an example which is worthy and might be followed with great benefit to the public at large by more pretentious cities.

Smallpox is epidemic in Americus, and the council passed an ordinance requiring every person to submit to vaccination. The city physician began the work. The christian scientists rebelled, declaring their faith was sufficient proof against the disease, and that they were not subject to the ills of ordinary mortals.

There was no arguing over the matter; no long-winded hearings before committees as in Buffalo. The city authorities promptly placed the scientists under arrest. Mayor Hixon assessed fines against them ranging from $3 to $30 and solitary imprisonment from ten to thirty days each. Five of the most prominent ladies of the city were sentenced to ten days' imprisonment and fined $3 each, the Mayor leaving it with the chief of police to select the place of confinement. They may be quarantined at their homes. A leading merchant was sentenced to thirty days in the city jail and to pay a fine of $30.

Many other scientists will be tried and like sentences will be imposed. All are technically charged with disorderly conduct. They have employed counsel and will take the cases to the highest court. The christian scientists declare that their religious freedom is being infringed upon and that the city has no power to vaccinate them. They say they will go to jail and rot before they will submit to the virus.

From the way Mayor Hixon has started in, it is safe to say scientists of Americus, Ga., will be vaccinated.

